# Supplementary figures and images for: It’s about time: Analysing simplifying assumptions for modelling multi-step pathways in systems biology
Source: PLoS Comput Biol. 2020 Jun 29;16(6):e1007982. doi: 10.1371/journal.pcbi.1007982 (PMC7351226; doi:10.1371/journal.pcbi.1007982)

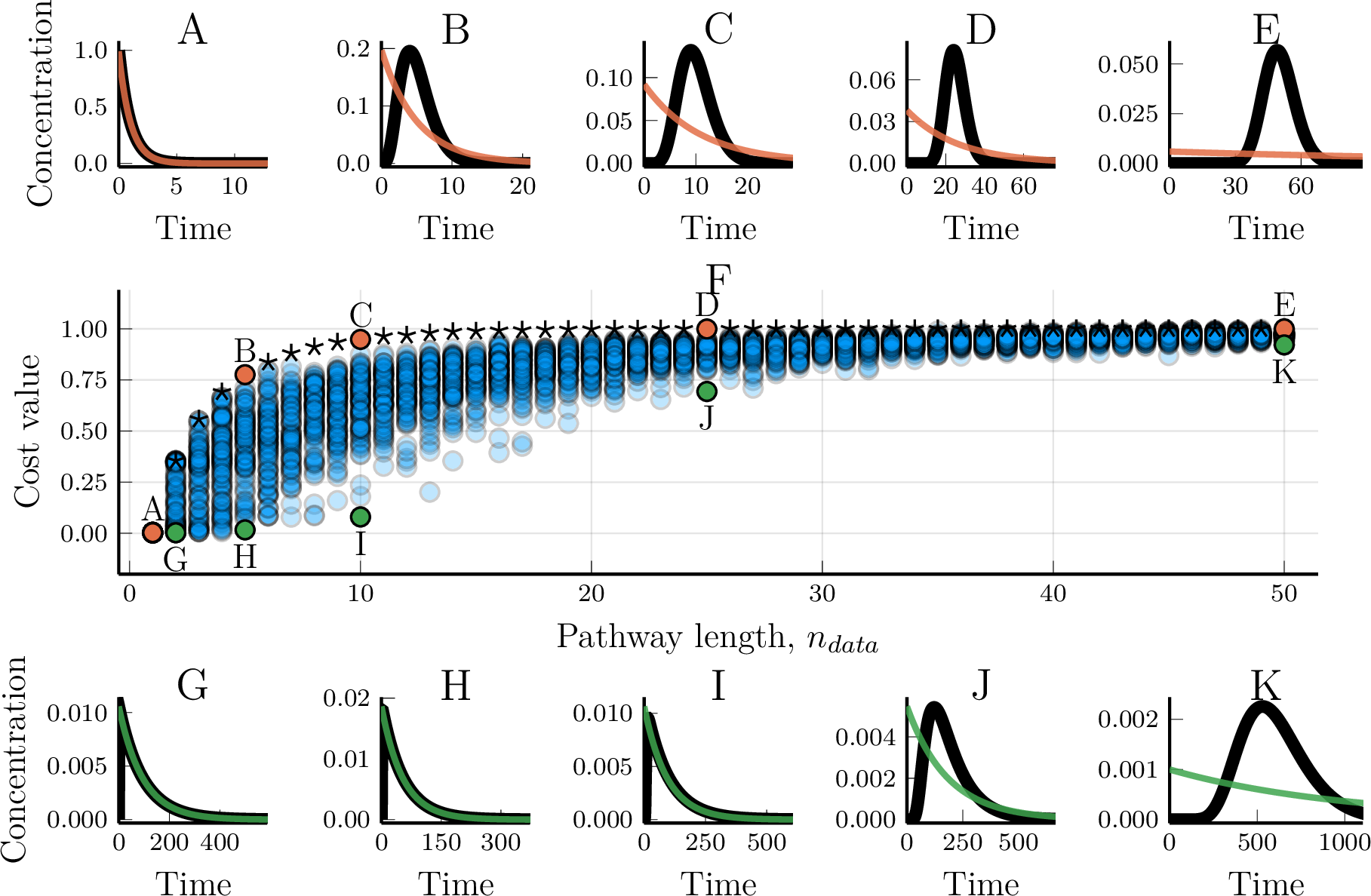

Supplement: S1 Fig — (A-E) The worst model/data fits for a given length, ndata, of the model that generated the data. Coloured lines show simulations of the fitted model while black lines show the synthetic data. (F) The cost value for 5000 parameter sets, each optimised towards a different set of synthetic data. Circles show the cost values resulting from data wherein all the steps in the data-generating linear pathway were randomly drawn, ri ∼ 10U(−2, 1) ∀i. Stars are the cost values from data wherein the pathway has homogeneous reaction rates, ri = 1 ∀i. The x-axis shows the number of steps in the model which were used to generate the data. (G-K) Examples of the best model/data fits for different data pathway lengths, ndata. (TIF) [file pcbi.1007982.s001.tif]

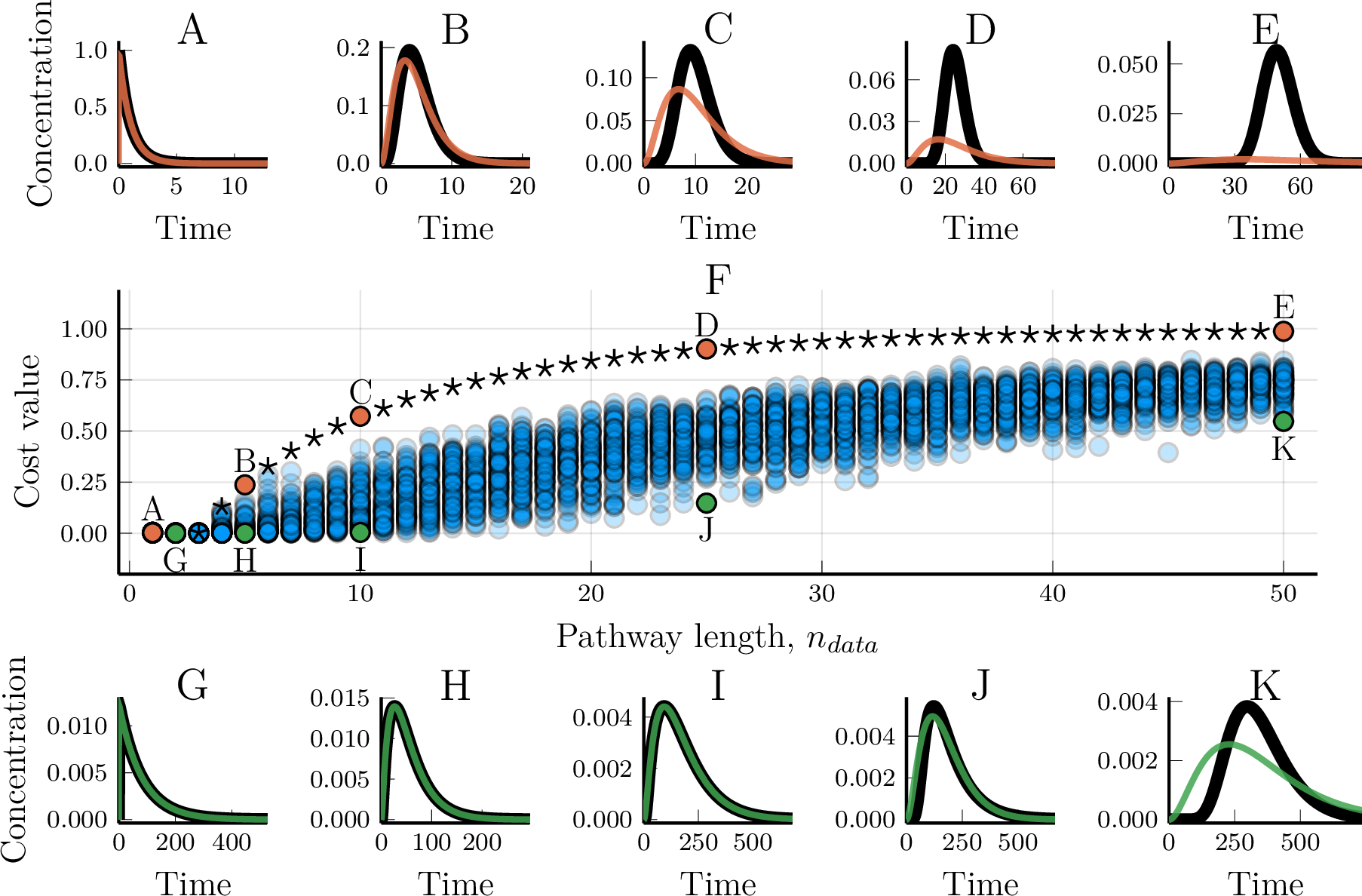

Supplement: S2 Fig — Description otherwise as in S1 Fig. (TIF) [file pcbi.1007982.s002.tif]

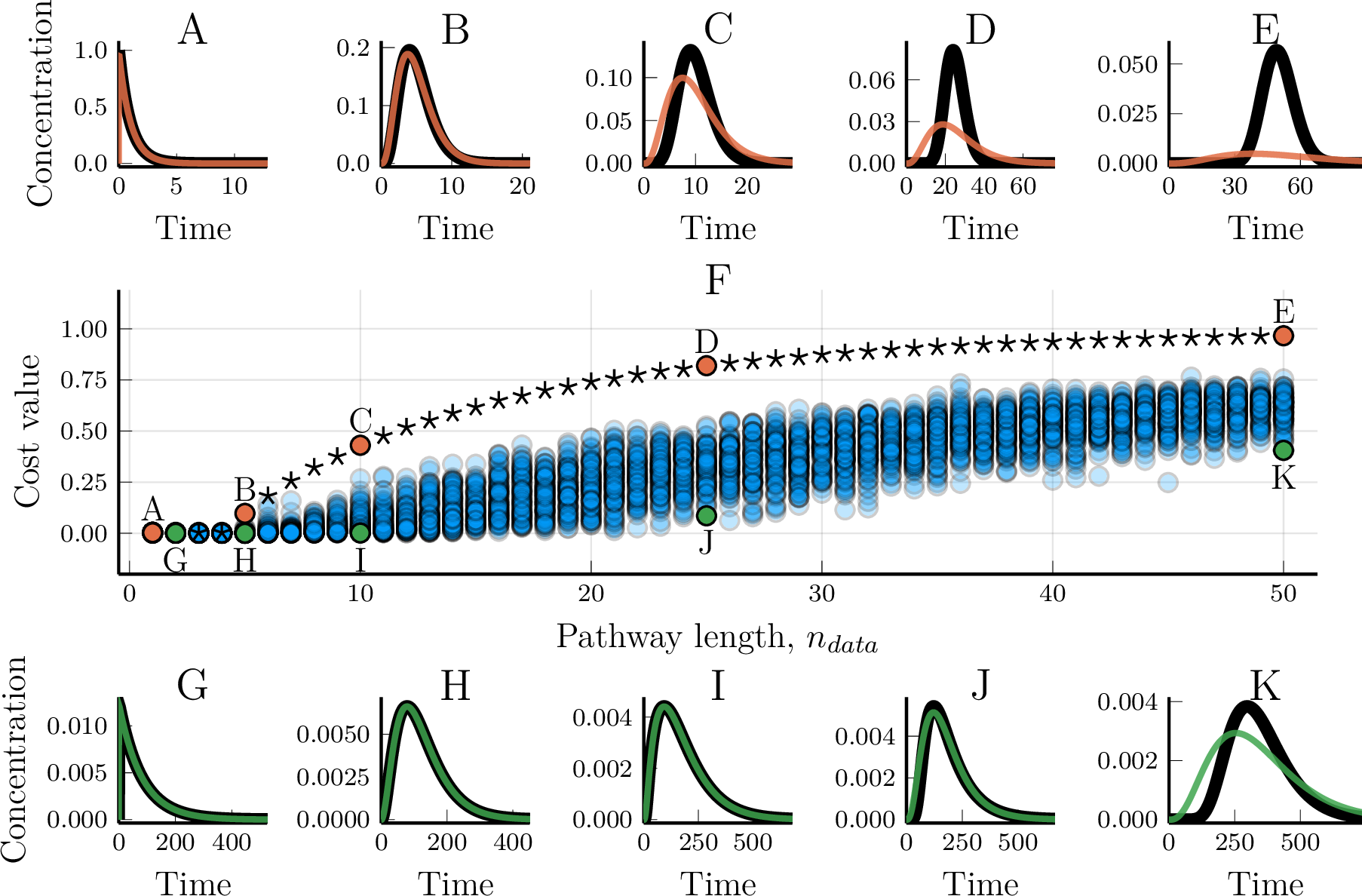

Supplement: S3 Fig — Description otherwise as in S1 Fig. (TIF) [file pcbi.1007982.s003.tif]

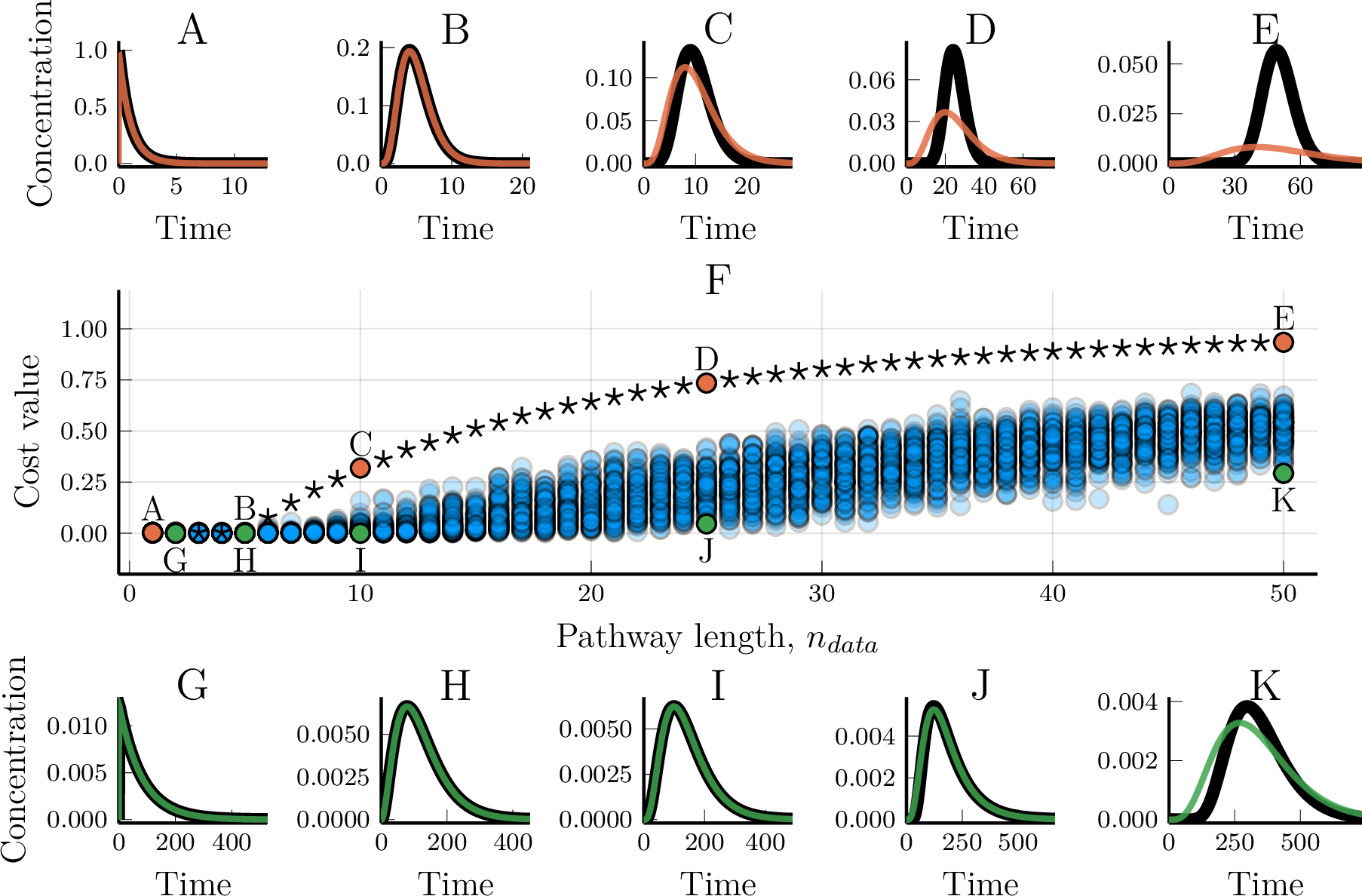

Supplement: S4 Fig — Description otherwise as in S1 Fig. (TIF) [file pcbi.1007982.s004.tif]

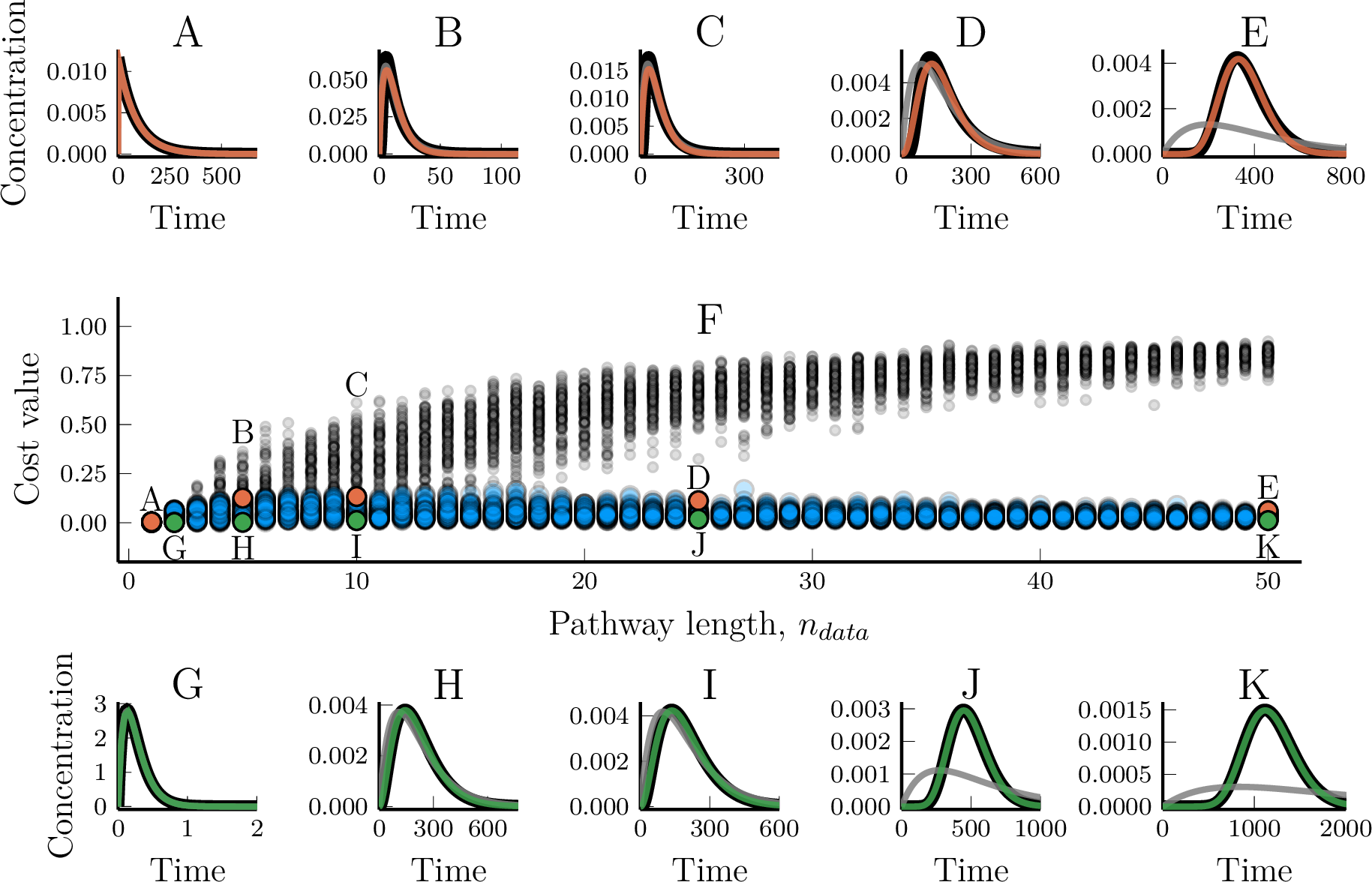

Supplement: S5 Fig — The gamma model (Eq 11) with nmodel∈R efficiently recapitulates linear pathway dynamics. (A-E) Examples of the worst model-data fits for different lengths of the data-generating pathway, ndata. Black lines show the synthetic data, orange lines show the results of the gamma model, and grey lines show the results of a two-step model (Eqs 2 and 3). (F) The ability of the gamma model to fit the data changes with the length of the underlying pathway, ndata. Blue dots show the cost value of the gamma model. The small, grey, dots show the corresponding cost values for a two-step model. (G-K) Examples of the best model-data fits. (TIF) [file pcbi.1007982.s005.tif]

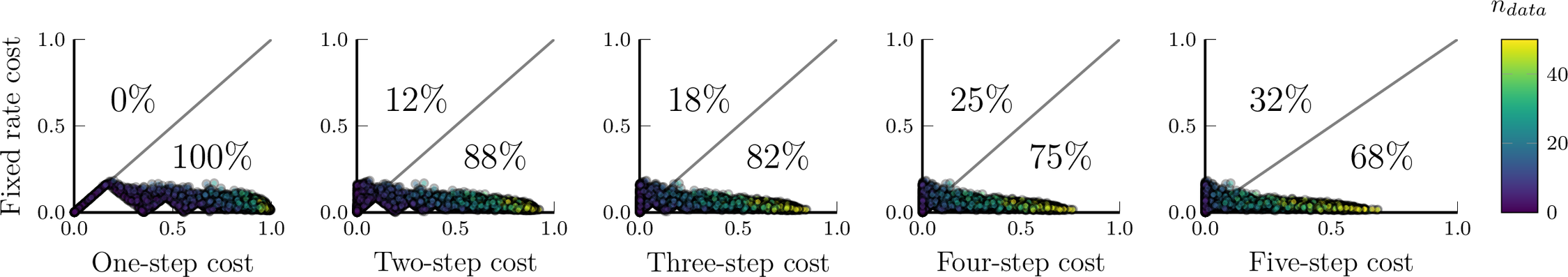

Supplement: S6 Fig — Each circle represents a single synthetic data set. 20 synthetic data sets were generated for each ndata ∈ {1, …, 50} using the impulse input (Table 1). The circles position indicates the optimised cost for the respective models and its color indicates the ndata value of the data-generating pathway. Percentages indicate how many of the data sets had a higher (worse) cost value for the respective models. (TIF) [file pcbi.1007982.s006.tif]
