# Supplementary material for: It’s about time: Analysing simplifying assumptions for modelling multi-step pathways in systems biology
Source: PLoS Comput Biol. 2020 Jun 29;16(6):e1007982. doi: 10.1371/journal.pcbi.1007982 (PMC7351226; doi:10.1371/journal.pcbi.1007982)
Supplement: Attachment — Submitted filename: review.pdf [file pcbi.1007982.s007.pdf]

Dear Editor,

we would like to thank the reviewers for their detailed and insightful remarks on the manuscript. We have tried to respond to all questions below. The resubmission has taken some extra time since we wanted to act on some of the reviewer suggestions and have accordingly extended the scope of the paper. In particular, we have (i) added and thoroughly analysed a non-linear case with saturating dynamics, (ii) added an example with real-world data to show how the fixed-rate assumption can be useful in practice, and (iii) formalised the analysis and discussion on when truncated models may have problems to further guide the reader on when a fixed-rate assumption is useful.

We feel we have substantially improved the manuscript with all changes and hope the reviewers (and readers) are further convinced about its merits.

Best regards,

Henrik Jönsson and Niklas Korsbo

Reviewer 1

General comment

In biochemical network modeling, it is a common practice to replace a long sequence of reactions with fewer reaction steps. Such simplification often involves lumping and rescaling of rate parameters to keep the reduced network consistent with the original system. This manuscript points out the caveats of such simplification and provides a few alternatives. The main analyses and findings of this work are summarized below.

The manuscript considers a linear pathway consisting of N reaction steps. It also considers another linear pathway consisting of n steps ($n \neq N$) that serves as a model for the N-step pathway. By fitting the n-step model to (synthetic) data produced by the N-step pathway, the authors investigate how well the model recapitulates the system's dynamic behaviors in response to several inputs. It appears that the model performs poorly when $n << N$ (most analyses involve N = 50, n = 2), especially when the steps are assigned with similar or identical rates. The authors then propose an alternative model, where n is treated as a free variable whose value is determined by optimization while the reaction rates of all steps are held fixed. In the majority of the cases, this alternative model (fixed-rate model) outperforms a two-step model. Finally, the authors provide an analytical form for the fixed-rate model for a simple case where the system input is zero. This analytical form, called the beta model, is not subject to the constraint that the number of steps n must be an integer. This beta model outperforms both the fixed-rate and the two-step model.

The analyses are interesting and the reviewer thinks the manuscript is well-written. Nevertheless, I have several questions, as listed below.

Thank you for these encouraging words!

Major comments

1.1 The equations indicate that the authors only consider first-order reactions in describing their linear pathway. However, most biochemical transformations (steps) are better described by the Michalis-Menten rate law. For example, a phosphorylation reaction involves a transient complex formation between the substrate protein and an enzyme (kinase). Such steps are prevalent in the examples provided by the authors in the introduction: MAPK activation, transcription or translation by RNA polymerase or ribosome, sequential protein phosphorylation in various pathways, etc. Could the authors provide examples of published models that treated these steps as first-order reactions?

Are the analyses and conclusions still valid if the Michaelis-Menten rate law is used to describe the steps in the sequential pathway?

We have expanded the scope of the paper to include strongly activated pathways (Michaelis-Menten type dynamics).

We hope that this expansion of scope both resolves this issue as well as helps convince the reader (and you) that the fundamental idea behind this manuscript is useful in practice and not just in our examples.

In the manuscript, we

- Added a new results section where we show that a fixed-rate assumption is often better at simplifying a non-linear cascade than a fixed-step assumption.
- Added the methods behind this analysis to Methods.
- Edited title, abstract, introduction and discussion to reflect the new and broader scope of the paper.

1.2 The analyses provided are based on generic or toy examples. Better if the authors provided a case study involving a specific biological pathway. Examples could be published models where such simplification was applied.

We included an example where we fit the two-step and the fixed-rate models to real data from plant immune responses. The following now ends the fixed-rate subsection:

To verify the applicability of the fixed-rate model beyond our synthetic data, we compared its performance with that of the two-step model when fitting real data. For that, we used data from the immune response of *Arabidopsis Thaliana*. There, a set of different surface receptors respond to different danger-associated inputs and then, through a multi-step pathway, trigger an output of reactive oxygen species (ROS) Couto and Zipfel [2016]. The ROS response is transient even though the input is persistent and this is hypothesised to be due to input-induced degradation of the receptor. We can interpret this as an initially inactive pathway where only the first step (the inactive receptor) has a non-zero concentration. At time $t = 0$,

the receptors start binding to the input and keeps on producing signalling until they are degraded. This case is analogous to the impulse input that we have used in the paper. The multi-step pathway leading to the release of ROS is not perfectly characterised and we do not know whether or not it is linear. We do, however, know that for one of these inputs, flagellin22 (flg22), there is a sequence of at least five reactions that occur before the production of ROS is triggered Couto and Zipfel [2016]. Fitting the models to this data clearly shows that the fixed-rate model is better able to capture the experimentally observed dynamics (Fig 7).

This is accompanied with a new figure (Fig 7 in the revised manuscript).

1.3 Most analysis involve a 50-step vs. a 2-step model (N = 50 vs. n = 2). A 50-step linear pathway without having an intermediate crosstalk/non-linear component/feedback seems unlikely in cell signaling or gene transcription. The result indicates the two-step model performs quite well when $N < 10$, and the two-step model is almost as good as the fixed-rate model in the range N =5 -10 (Fig. 4). In a more realistic scenario, where $N < 10$, is the two-step model adequate?

For the performance question for different scenarios for the truncated models we refer to **1.4**.

Regarding the choice of the maximal pathway length, we would think that there are (at least) two classes of pathways. 1) Heterogeneous pathways such as signal transduction, cascades, sequential conformation changes or gene regulatory 'pathways' are not likely very long or highly connected with feedbacks (as pointed out by the reviewer). 2) Homogeneous, repetitive things such as active transport along a microtubule or file of cells, or transcription/translation are however often much longer than that. We set the maximum number of steps to 50 because this is when the two step-model fairly consistently failed and the inclusion of even longer pathways seemed to obfuscate the plots we made. The failure of the two-step model for even longer pathways can easily be inferred from this and the success of the model for shorter pathways can be directly seen from the figures since they often have the underlying pathway length as an axis.

1.4 The optimization evaluated the number of steps for the fixed-rate model. This should lead to many more steps in the fixed-rate model compared to the two-step model when N = 50 (Fig. 5 and 6). Is not it expected that the fixed-rate model outperforms the two-step model because the former involves more steps? The analysis shows that the fixed-rate model outperformed the 2-step model in approximately 90% of the cases. How would this result change if the fixed-rate model was compared against a 5-step model? Or if the data was generated using a 10-step pathway (N = 10)?

We thank the reviewer for pointing out that the performance of truncated models in different scenarios was not fully clear in the previous version of

the manuscript. We want to point out that the analysis in not solely for a N=50 pathway but for pathway lengths up to N=50, and for different inhomogeneity in parameter values for individual pathway steps, since we found these parameters (length and homogeneity) are highly relevant for performance, and we do compare different truncation lengths in the Supplemental Figures (- to be compared to the fixed-rate case, Fig. 5).

The question of which model is better does not only depend on the length of the approximated pathway but also on the rates of signal transmission along that pathway. To more clearly show when one model outperforms another, we provided new plots that map out this pathway length - parameter inhomogeneity space (Fig. 3g-i). These are heatmaps that show the cost for (and ratio between) the fixed rate and the two-step models for different n_{data} and for different degrees of parameter inhomogeneity. This clarifies that the answer to your question is actually multi-dimensional while also providing fairly concrete answers about which model is better under which condition. More explicitly, a 10-step pathway can be represented fairly well by a two-step model only if the transfer rates are highly heterogeneous (Fig 1i). The model will, however, perform poorly for more homogeneous transfer rates (Fig 1c). We also want to stress that e.g. a five-step model has twice as many free parameters as the fixed-rate model. This could also be taken into account when evaluating the effectiveness of a simplification but that would add complexity. Instead, we focused on truncated models of length two since they have the same number of free parameters as the fixed-rate model.

We recognise that our way of communicating the relative performance between the models was a bit too absolutist and we modified the paragraph that compares the fixed-rate and the fixed-step models to reflect this more nuanced story. We think that the manuscript has benefited from this.

As for whether it was expected that a fixed-rate model, with more variables, would outperform the fixed-step model, we would say that it is not automatically so that more variables increases a model's dynamical range. The fixed-rate model in its ODE form has n internal variables but its analytical solution has none. We argue that a better predictor of a model's degree of freedom is the number of free and linearly independent parameters it has.

1.5 "—- it is easy to find examples where it has been used to simplify multi-step reactions such as protein production (e.g. 7, 31, 33); protein-to-protein signalling networks (e.g. 7, 32, 34); protein modifications such as 57 phosphorylations, methylations, and ubiquitinations (e.g. 33, 34)"

— It seems like these simple models (cited references) did not truncate or simplify a linear multistep reaction, rather the goal could be to use a minimal model to describe specific data or experimentally-observed phenomena. Are these citations relevant in the context of this work?

These citations serve only to demonstrate that unbranched multi-step reactions are indeed simplified in some published papers. It is a rather trivial point but it still works as motivation for our work since our results could have been useful during the creation of these models. The simplifications in these models are all rather implicit and we cannot claim whether the bits that were reduced away were linear. We do also refer to models where fixed-rate models have been used in practice e.g. for circadian oscillations Tokuda et al. [2019], and now provide an explicit example with real data showing that the fixed-rate model allows for a better model-data fit than the two-step model (cf. our reply to **1.2**).

Minor comments

1.6 In the DDE model, the delay time is enforced in the final step. Does it make any difference if the optimization is allowed to choose where to introduce the delay time? What if more steps are allowed with fixed or heterogeneous delay times?

This was a fun question and we'll give you the answer. We did not, however, include it in the manuscript since we felt that our attempts to insert it always seemed contrived and a bit beside the point.

The position of delays in a fixed-delay model does not matter for the trajectory of the output. Nor does it matter if delays are broken up and distributed across any intermediate steps. Such fixed delays only serve to statically delay the output curve, without affecting the shape thereof. The total delay contributed is just the sum of all such fixed delays. Thus, only the sum of the delay matters, not their order nor how many separate delays they are split into.

This can be proven by looking at the transfer function from the input to the output of a system of n steps, all of which having individual delays, τ_i, in their response to the previous step,

$$\begin{aligned}\dot{X}_1 &= r_1 \cdot (I\,(t-\tau_1) - X_1)\\ \dot{X}_i &= r_i \cdot (X_{i-1}\,(t-\tau_i) - X_1)\end{aligned}$$

with a unit impulse input $I\,(t) = \delta\,(t)$, $X_i(0) = 0 \quad \forall i$ and delays τ_i.

From here, we apply the Laplace transform, $\mathcal{L}$, on both sides of the equations, utilising the fact that the Laplace transform of a function derivative, f', follows $\mathcal{L}[f'] = s\mathcal{L}[f] - f(0)$. This leads to

$$\begin{aligned}s\mathcal{L}[X_1] &= r_1 \cdot (\mathcal{L}\,[\delta\,(t-\tau_1)] - \mathcal{L}[X_1])\,,\\ s\mathcal{L}[X_i] &= r_i \cdot (\mathcal{L}[X_{i-1}\,(t-\tau_i)] - \mathcal{L}[X_i])\,,\end{aligned}$$

where $\mathcal{L}[\delta(t-\tau_1)] = e^{-s\tau_1}$ and $\mathcal{L}[X_i(t-\tau_i)] = e^{-s\tau_i} \cdot \mathcal{L}[X_i(t)]$.

The set of equations can thus be rearranged to get

$$\mathcal{L}[X_1] = \frac{r_1}{s+r_1} \cdot e^{-s\tau_1},$$
$$\mathcal{L}[X_i] = \frac{r}{s+r} \cdot e^{-s\tau_i} \cdot \mathcal{L}[X_{i-1}].$$

This can be recursed to solve for n steps, leading to

$$\begin{aligned}\mathcal{L}(X_n) &= \prod_{i=1}^{n} \left(\frac{r_i}{1+s} \cdot e^{-s\cdot\tau_i} \right) \\ &= \prod_{i=1}^{n} \left(\frac{r_i}{1+s} \right) \cdot e^{-s\cdot\sum_{i=1}^{n}\tau_i} \\ &= \prod_{i=1}^{n} \left(\frac{r_i}{1+s} \right) \cdot e^{-s\cdot\tau_{total}}\end{aligned}$$

where we see that only the total duration of all delays matter, not their order nor their distribution along the pathway.

1.7 Font sizes in the legends of Fig. 5d and 6E are too small to read.

We thank the reviewer for pointing this out. We have updated the legends accordingly.

1.8 In the result section, figures are often referenced randomly without following any specific order.

We have followed the rule to number Figures following the order when they are first mentioned in the text. We have split Figure 2 into two separate figures and rearranged the sub-figure order so they better follow the order in which they are mentioned in the text. For others, they mostly compare different models and we argue it is better for a reader to have the different models in the same figure to facilitate the comparison, but we are aware of the trade-off for needing to go back from the text where new models are introduced. We also expect this will become better in a real-layout paper where everything is more compressed compared to the preprint version.

Reviewer 2

This paper focuses on the impact of number of reaction steps in linear biochemical pathways. They introduce a framework for linear pathway simulations in order to compare several reduction operations over such pathways : reduction of the number of reaction steps, introduction of several classes of delays. Based

on their simulations on synthetic dataset, the author conclude that simplifying to a three-model parameters (more precisely, scaling, pathway lenght, homogeneous response rate of the pathway) outperforms both state-of-the-art reduction approaches.

The paper is well-written, interesting and technically correct. However, I have doubts on its applicability on real models.

Thanks for the encouraging words! We also hope that we now have addressed the matter of applicability to a point where the practical usefulness of these models are evident.

2.1 The first issue raised by the paper is that the main assumption of the author is that is a linear chain of reactions can be modeled with a linear differential system, without taking into account any non linearity effects. The study performed by the authors concerns only the biological systems for which this assumption is true. The paper would be more convincing if the author could detail the number of biological models (for instance in the BioModels database) for which this linear model assumption is used.

We thank the reviewer for pointing out the concerns on usefulness, and we have now addressed this in several ways (several are discussed in the responses to Reviewer 1 questions).

We think that it is fair to be worried that the presence of non-linearities in biological pathways exclude the application of models based on purely linear pathways, and we have added a section that shows that the fixed-rate assumption can be used to good effect for non-linear cascade models (see our reply to **1.1**). We still would like to stress that also linear pathways are of importance in several biological contexts (see our reply to **1.3**).

To show the applicability to a realistic case, we applied the linear models to some real experimental data (see our reply to **1.2**). We think that their success in capturing the observed dynamics does indeed show that the models can be useful.

We have refrained from investigating a subset of models from e.g. the BioModels database since we argue it would require some extensive analysis in terms of exactly what the data sets available are and what assumptions have been made in individual cases. As mentioned in the paper, there is an example of this already in the literature Tokuda et al. [2019], where the fixed-rate assumption has been applied successfully but where no analysis of a general approach allowing pathway length as a free parameter has been applied.

We argue that the two additions of non-linear analysis and a real-data example together with the literature examples strengthen the evidence of an extended applicability and we can only hope that you would agree.

2.2 A second issue is that the synthetic data used to conduct all the experimentation were generated according to a linear ODE model with different pathway lenghts (from 1 to 50) and a log-uniform distribution for response

rates. How valid are these hypotheses with respect to existing ODE models of biological processes processes in the litterature ? Simulating several linear published models to provde that the synthetic data are realistic seems necessary to validate the approach.

The broader meaning of this concern seems to be one of applicability of our models. We have addressed this by increasing the scope of the study to encompass non-linearities (see Response **1.1**); showing that the results hold for any input, including feedbacks (see Response **2.3**); and applying the theory onto real biological data (see Response **1.2**).

We do appreciate the Reviewer's concern that we did not well enough describe that the generated data do encompass a large dynamic range of linear pathways which makes it a good illustration of when and why truncated models can be problematic. As discussed in Response **1.4**, we have now added plots to span the pathway length vs parameter inhomogeneity to better display this. We argue that these carefully selected parameter regions include what we would expect to find in reality. In addition (see Response **3.9**) we made a more rigorous analysis of what dynamics different linear models are able to produce. From that analysis, we hope that it is fairly clear that the cases we studied do cover a very large portion of those possibilities. Similarly, in the added section on non-linear models, we identified the 'level of saturation' as an important feature for the applicability of different models, and included an analysis of the inherent problem for non-linear systems of predictability given dynamical ranges for changes in input levels.

We appreciate the Reviewer's suggestion to use examples as proofs of validity as a complement our approach, but argue that our more general investigation is highly useful for an expert on a specific system to evaluate when it is appropriate to try a fixed-rate assumption. Again, we would like to explicitly highlight Tokuda et al. 2019 as a recent example of this. There, a gamma distributed delay (which assumes both linearity and a fixed rate) is used to simplify several models of circadian rhythm without losing model/data agreement. They make a considerable effort along these lines so we feel that we cannot do this better than they already have, but note that if they had started by using the pathway length as a free parameter as we suggest, they would have avoided the 'ad-hoc' trial and error of trying different pathway lengths.

This last point is now mentioned with more emphasis in both the introduction and the discussion.

Intro:
Furthermore, it was recently shown to be effective at simplifying specific models while still allowing them to retain the dynamical properties of the original models Tokuda et al. [2019].

Discussion:
Here, we argue for its effectiveness by applying it to both synthetic and

experimental data and we argue that it is applicable for any model input. This was neatly exemplified in Tokuda et al Tokuda et al. [2019] where they showed that introducing distributed delays (fixed-rate assumption) between components of circadian clock models allowed for a parameter reduction while retaining the main dynamics.

2.3 A third issue is that, as mentionned by the authors, linear pathways occur in biological models as combined with self-regulatory controls that may impact on the input signal. Therefore, the model of the input signal should deserve a very special attention to validate the conclusion of the authors. Addressing this issue could be done for instance by applying the three reduction methods studied in the paper on a family of complex published and validated models in order to figure out if the conclusions are still valid for input functions controlled by the system dynamics.

We have added a mathematical argument for why our conclusions for the linear pathways are valid for any input, including inputs that are affected by the output of the pathway itself.

The "none"-input that we previously described is equivalent to an impulse input. The impulse input response is the inverse Laplace transform of the transfer function which means that it fully describes the transformation that the model does to an input signal before passing it on as an output. This is useful since if two linear models have (approximately) the same impulse response then they will also perform (approximately) the same transformation of their input to their output, regardless of what that input is. Thus, if a simplified model can reproduce the dynamics of the full system under these conditions, it will be able to reproduce the dynamics of the system for any input function, including inputs that depend on the feedback from the output of the linear pathway. In response to your question, we decided to incorporate this in the manuscript by re-framing the narrative around the "impulse input" and also explain its special dynamical relevance.

This change affects the manuscript in many places. Most of it was just trivial edits but the main change occurred in the paragraph where we first describe the model input which now reads:
To best characterise how well a model could perform when $n_{model} \neq n_{data}$ we focused on studying their response to a unit impulse input, $I(t) = \delta(t)$, from an initially inactive state, $X_i(0) = 0 \; \forall i$. This input is useful since analyses of the impulse response is easily extendible to arbitrary inputs Ogata [2010] (Methods). It is worth noting that the impulse input is equivalent to the pathway receiving no input at all but instead start its simulation from an initial condition where $X_1(0) = \gamma r_1$ in an otherwise fully inactive pathway. This simulates the sudden start of a reaction at $t = 0$ where X_1 passes on its signalling while being exponentially depleted itself and could represent the conversion of a depleting responder upon the onset of an external signal.

Due to the mathematical equivalence of the 'none' input and the impulse input, this reformulation did not affect on any results, only the reporting thereof.

With this addition, a part of the discussion where we mused that the effects of the linear pathway simplifications in complex networks would be interesting to study became obsolete and was thus removed.

2.4 Finally, the author advocate that a software is associated with their publication. It appears that this software is a code in the Julia programming language allowing to reproduce the simulations shown in the paper. It should be clarified that the software is not a tool for model reduction (and parameter fitting) as it could be expected while reading the paper.

Our intention was never to provide a general software package for model reduction and parameter optimisation. However, we do argue it is highly important to provide the software used to produce scientific results for anyone else to reproduce these results or develop methods further. To clarify this, we changed

The source code developed for this project is openly available under the MIT licence at the Sainsbury Laboratory GitLab repository https://gitlab.com/slcu/teamHJ/publications/Korsbo_et_al_2019.

to

Source code for the reproduction of the results is openly available under the MIT licence at the Sainsbury Laboratory GitLab repository `https://gitlab.com/slcu/teamHJ/publications/Korsbo_et_al_2019`.

Reviewer 3

The manuscript addresses the problem of the number of steps in a simplified, linear model of signaling with no branching. This is an important issue and may guide the modeler's choice. The main result of the paper is that a "fixed rate" model with a variable number of steps is well suited for fitting a broad corpus of data. This idea has already been used in the systems biology literature but never tested systematically. The submitted work has the merit of testing this reduction ansatz. Nevertheless, the conclusions on the applicability of the "fixed rate" ansatz are entirely based on numerical simulations instead of precise mathematical estimates. In many places, the manuscript can be substantially improved by precise definitions and more rigorous specification of the domain of validity of the results. A list of issues that need major amendments follows:

3.1 The idea to use the number of steps as a free parameter has already been used for a chain model of transcription in Dufourt et al Nature comm. (2018) 9: 5194, where the authors chose the number of steps and the homogeneous or heterogeneous rates on the bases of quality of fitting and variability of optimal and suboptimal parameters; for the optimal number

of steps a heterogeneous rate hypothesis is rejected on the basis of parameter variability. These similar ideas should be cited in the manuscript.

We thank the reviewer for pointing out this paper as an example where the approach has been used. We were not aware of this paper but we have now added a reference to it in the introduction.

While similar ideas have recently been used, their effect has not to our knowledge been systematically studied before Dufourt et al. [2018], Tokuda et al. [2019].

3.2 The type of model and the meaning of linearity should be made more precise in summary and introduction. Which type of models are dealt with, stochastic or deterministic? The authors use the name "linear pathways" to speak of a special case of first order chemical reaction networks (CRNs), first introduced by Heinrich et al (2002) ref. [12]. In this context linearity may mean both "first-order" and unbranched topology.

We agree that the term "linear pathways" is was ambiguous so we updated the manuscript to clarify the issue. Parts of these updates also came naturally due to the expansion of scope to include non-linearities.

The term "linear pathways" is no longer present in the title.

In we abstract, we now write:
We first focus on linear pathways that are sequential and have first-order kinetics

In the introduction, we write:
Here, we examine the dynamical effect of sequential multi-step pathways on a system and especially whether certain simplifying assumption yields models capable of reproducing those dynamical effects. We primarily focus on linear pathways—where each step is linearly dependent on the last—where we first analyse what dynamical properties a model will be unable to reproduce when it is simplified using pathway truncation.

We, thereafter, continue to use "linear pathways" since greater specificity often results in sentences that are harder to read.

3.3 The notion of "step" is crucial in the paper and needs careful discussion.

We agree that this notion is central and important for the study, but we have the impression all reviewers' use of the word indicates that the concept has been understood from our definitions. Also, whether a 'step' refers to the nodes or the edges of the pathway graph does not actually change the way it would be used in the manuscript. However, we have thought of it as nodes rather than edges. We have now tried to clarify this further in a way that does not assume the reader to know graph-notation.

To investigate this, we first defined a model wherein a sequence of n states (which we will also refer to as steps), with concentrations $X_1, X_2, \ldots, X_n$, each activates its successor.

3.4 It is not true that little effort has been made to understand pathway truncation. Mathematical theories of pathway truncation are available for monomolecular CRNs (Gorban and Radulescu Adv.Chem.Eng. (2008) 34:103), first order CRNs (Helfferich J.Phys.Chem. (1989) 93:6676), and more general CRNs (Radulescu et al, Front. Genet. (2012) 3:131).

We were not aware of this good and relevant body of work and we thank the reviewer for pointing it out. We have now rephrased the introductory paragraph on pathway truncation to better reflect what has been done and what knowledge gap we seek to fill.

A common way of simplifying linear pathways is to ignore most of the reaction steps and assume that a model can recapitulate their effect using only one or a few steps [Aldridge et al., 2006]. Such topological model reduction is common and the approach has been rigorously analysed for both simple and complex networks Gorban and Radulescu [2008], Radulescu et al. [2012], Helfferich [1989]. While important, such analyses often presuppose precise knowledge of the system that is being simplified. Nevertheless, while this assumption is often implicit, it is easy to find examples where it has been used to simplify multi-step reactions in real systems—where many details are unavailable—such as protein production (e.g. Hausser et al., 2019, Collier et al., 1996, Gruel et al., 2016, Gordon et al., 2009); protein-to-protein signalling networks (e.g. Collier et al., 1996, Gruel et al., 2016, Gordon et al., 2009, Barkai and Leibler, 1997); protein modifications such as phosphorylations, methylations, and ubiquitinations (e.g. Gordon et al., 2009, Barkai and Leibler, 1997); and more. A question that remains is then what dynamical behaviour a model is prevented from reproducing when this kind of simplifying assumption is applied to partially unknown systems.

3.5 The scaling leading from Eqs (1) to (2) and (3) should be explicitly given in the main text (this is not available in the methods).

This is remedied in our reply to **3.15**.

3.6 Clearly specify that the output is Xn(t).
Good point. We clarified this during the initial model description by explicitly stating that:
Where ... and $X_n(t)$ is the output of the pathway.

3.7 Eq. at line 120 (now line 136) is valid only if $Xi(0) = 0$ for $i > 1$.

This is true but the point is now moot since the equation was removed when we changed the input-describing paragraph in response to question **1.2**.

3.8 At various places fitting is said to be guaranteed perfect. But fitting results from a numerical scheme; even if theoretically a perfect fit is possible, the scheme may not find it. At other places fitting is said to perform well.

What does this mean quantitatively and on which statistical grounds?
We thank the reviewer for pointing out our misuse of language.

It is true that we cannot claim the numerically optimised parameters to be optimal. We, therefore, replaced inappropriate references to "optimal" parameter with references to "optimised" parameters.

Around line 166, we also replaced:

When the fitted model has fewer steps than the linear pathway which was used to generate the data, $n_{model} < n_{data}$, the fit is no longer guaranteed to be perfect.

with

When the model has fewer steps than the linear pathway that was used to generate the data ($n_{model} < n_{data}$) it may no longer be possible to find a good fit.

As for the other part of your question: the parameter fitting does well by a few different measures. First, they to do well upon inspection of the resulting dynamics. Second, they lead to the expected smoothness of how the parameter or cost values changes with the change of different constraints, as seen in many of the figures. Third, models that we know to be able to perfectly represent the data do indeed fit very well and the optimized parameters reflect the parameter values that generated the data. Fourth, the fitted gamma model lead (in almost all cases) to lower cost values than what you get analytically by demanding that the mean and variance of the signalling delay is the same for the model and the "data". We cannot prove that the optimisation scheme yields approximately globally optimal results but we have indeed been mindful of this issue and we have performed multiple tests and sanity-checks throughout our work to ensure reasonable solutions and that our conclusions are not a result of failed optimisations.

3.9 Some criteria are proposed to identify "detrimentally truncated models", such as the amplitude and width if the output signal and the variability in the rate parameters. This section is important and should be treated with more care. What quantitative recipes should be used here?

We thank the reviewer for pointing this out and agree completely. We took some time to investigate this further and we think that the revised manuscript benefits greatly by it.

We rewrote that section, adding mathematical definitions of signal delay, duration and sharpness and showing how this leads to an inequality which bounds signal sharpness by the pathway length. We elaborate on how this sometimes renders the fixed-step model unable to reproduce data. We also show how homogeneous parameters lead to the maximal possible signal sharpness and how models tend towards this solution when the data at hand is more sharp than the model can account for. We also split up what used to be figure 3 into two separate figures. What used to be Fig 3a was

expanded upon in what is now Fig 4 and this helps demonstrate the points we are making.

We did not include a quantitative recipe for modellers to use. Firstly because we think that the concept itself is sufficiently simple for researchers to be able to make practical use of it without further guidance. Secondly, we fear that we would never be able to catch all use-cases (or exceptions thereto) with such a recipe. We would, therefore, rather see that individual researchers motivate their methods with our results rather than blindly following methods proposed by us.

3.10 The eq (6) corresponds to a singular delay model, whereas the fixed rate model leads to a distributed delay model. This should be clearly stated. The explanation of the failure of the singular delay model (lines 218-220, now 289-296) is obscure.

We had already referred to the connection between both the fixed-rate model and the gamma model to a gamma distributed delay.

The introduction stated and still states that "The [fixed-rate] assumption allows for a direct derivation of the gamma distributed delay"

We had also already referred to the gamma model as a gamma distributed delay but we have now made the point more clearly. We had previously wanted to avoid emphasising the value of the impulse-response function since might make the paper harder to digest for modellers who have less mathematical backgrounds. We reversed this decision in response to **2.3** and as an added benefit, we could make the gamma model section both more terse and precise (at the cost of requiring more from the reader). We are now more explicit about the gamma model being a distributed delay and the best example of this is the added phrase:

This is done through a convolution and because $g(t)$ is really identical to the probability density function of the gamma distribution (scaled with γ) the result is called a gamma-distributed delay,

We also refer several times to the equivalence between the fixed-rate model and the gamma model with integer n. We thus think that the connection between this model and the fixed-rate ODE model is clear enough for it to be obvious that it is equivalent to a distributed delay.

We had not used the phrase singular delay for the fixed-delay model but have now added such a reference for clarity.

In this 'fixed-delay' model, the pathway step that we consider an output, X, responds to an input at a rate governed by r, with a fixed, singular, time delay, t_{delay}.

We also clarified what the fixed-delay model does well and what it does poorly.

The fixed-delay model is able to adjust its signal delay (eq. 4) without affecting the signal duration (eq. 5). This allows it to fit the delay, duration,

and thus, sharpness of the data well. However, it fails to fit the symmetry of the output curve around its mean delay (skewness if we continue the distribution analogy). The model output starts very abruptly at $t = t_{delay}$ and its response to sudden input changes lacks the smoothness that is seen in the data. The fixed-rate model, on the other hand, can achieve the correct time-delay and sharpness while also accurately smoothing out the signalling over time.

3.11 The r parameter of the fixed rate model is said to represent the slowest step. This is rigorously true for separated rates but not necessarily true for similar rates. I don't think that Fig4 represents a rigorous proof of this correlation. Computing the correlation with next slowest steps and with the harmonic mean and comparing them with the slowest step would be a proof.

This is entirely correct. We still think that the relationship between r and $\min_i(r_i)$ is good for attaining some intuition about the problem but we now more accurately call this relationship a heuristic.

This question prompted us to investigate further and we found more precise mappings between the model parameter values and the underlying data. This applies not only to r but also to n. It also applies more precisely for the gamma model than the fixed rate model. We thus both moved the entire section to below that of the gamma model and we rewrote most of that section to include this more precise mapping between the (data-generating) system and the gamma/fixed rate model.

Apart from these changes, we also did as suggested and tested the correlation of r with both the second slowest rate and the harmonic mean of the rates. The correlation is pretty bad for the second slowest rate and for the harmonic mean the correlation is similar to that of the slowest rate. This inquiry led us to a better answer, written in the manuscript section. We did not, however, find it relevant to also include these investigations in the manuscript.

3.12 A gamma model with real shape parameter is proposed. This implies non-integer number of steps. What does it mean?

Both integer and non-integer values are often used in systems biology, even for parameters derived from discrete events (e.g. hill coefficients in Gardner et al. [2000], Elowitz and Leibler [2000]). In our case, as is now discussed more in detail in the paper (related to response **3.11**), there is not a precise coupling between the pathway length of the approximated and the n parameter. That coupling was already broken by the use of a simplifying assumption and allowing for real valued n simply improves the model's ability to fit the data.

3.13 The way how the restriction on the zero initial concentration can be lifted (lines 297-300) is not clearly explained.

We clarified what we mean in the methods, just before we apply the procedure to the wave input.

However, since we in the definition of the model assumed an all-zero initial concentration of the pathway, it is built-in to the model that it starts from an all-zero state. This limitation can, however, often be circumvented by performing the simulation in two stages. The first stage would apply the input required for the model to get to the proper 'initial' conditions for the second stage which simulates the model with the input one actually wished to study. For the wave input in this paper, this was achieved by using the input function

3.14 In the conclusion (also in the introduction) it is said that wrong delays can destabilize oscillations. To which extent is this true? Can the authors provide examples where delays with the same mean, but different distributions have different effect on the stability of oscillations? Some results from delay differential equations could be invoked.

We added a reference to Rombouts et al. [2018] which investigates the effect of different delays on oscillatory systems.

3.15 Methods should be proofread. Eq.11 seems to be used for proving a number of statements that are by no means clear. What is the meaning of the linear dependence of the production terms (line 380)? The whole paragraph between lines 378 and 390 is obscure. Same for lines 438-443, 447-450 (the l1 norm does not have the same advantages?). What are the "interpolations" at line 452?

We have reworked the methods for better clarity, paying special attention to the parts that you specifically mentioned as problematic.

You are correct that the integral cost is indeed an $\ell 1$ norm, just not the vector norm that is often used for cost functions but the function norm. In our cases, the difference is merely how we decide to sample the data. We updated the text to reflect this.

The interpolation we talk about allows the treatments of our ODE solutions (and the newly added biological data) as functions. We used standard interpolation methods (provided in DifferentialEquations.jl for the ODE solutions and linear for the data), and given the number of time points used to calculate the interpolations in both cases, any numerical errors generated by this step can be expected to be small and not affect any results.

Overall, we think that the information in the methods is sufficient to qualitatively reproduce our results and we hope that this information is now more digestible. For exact duplication, we would refer to the code that we share.

3.16 How is the heterogeneity parameter delta defined for the general distribution of rates? The definition in the figures is based on equidistant rates in log scale, which is a very special choice.

The heterogeneity parameter is defined in the figure legend and it is only used where indicated by a plot axis. We defined it to have a deterministic way of tuning the degree of rate homogeneity for this specific analysis, since we identified the inhomogeneity as an important parameter for performance (see e.g. Response **1.4**). We agree that it is a special case but it serves well and it is clearly defined. Rate heterogeneity for the other figures results from drawing the parameter values from a distribution (as stated around line 582) and their values are not governed by this inhomogeneity parameter.

Additional questions

Have all data underlying the figures and results presented in the manuscript been provided? Large-scale datasets should be made available via a public repository as described in the PLOS Computational Biology data availability policy, and numerical data that underlies graphs or summary statistics should be provided in spreadsheet form as supporting information.

Reviewer 1: None

Reviewer 2: Yes

Reviewer 3: No: The conclusions of the manuscript rely heavily on numerically generated data. This data should be made available on a public repository such as zenodo, for instance.

The code is indeed already available in a public repository, under a permissive license, as stated: "Source code for the reproduction of the results is openly available under the MIT licence at the Sainsbury Laboratory GitLab repository `https://gitlab.com/slcu/teamHJ/publications/Korsbo_et_al_2020`."

References

B. B. Aldridge, J. M. Burke, D. A. Lauffenburger, and P. K. Sorger. Physicochemical modelling of cell signalling pathways. *Nature Cell Biology*, 8(11): 1195–1203, 2006. doi: 10.1038/ncb1497.

N. Barkai and S. Leibler. Robustness in simple biochemical networks. *Nature*, 387(6636):913–917, 1997. doi: 10.1038/43199.

J. R. Collier, N. A. Monk, P. K. Maini, and J. H. Lewis. Pattern Formation by Lateral Inhibition with Feedback: a Mathematical Model of Delta-Notch Intercellular Signalling. *Journal of Theoretical Biology*, 183(4):429–446, 1996. doi: 10.1006/jtbi.1996.0233.

D. Couto and C. Zipfel. Regulation of pattern recognition receptor signalling in plants. *Nature Reviews Immunology*, 16(9):537–552, 2016. doi: 10.1038/nri.2016.77.

J. Dufourt, A. Trullo, J. Hunter, C. Fernandez, J. Lazaro, M. Dejean, L. Morales, S. Nait-Amer, K. N. Schulz, M. M. Harrison, C. Favard, O. Radulescu, and M. Lagha. Temporal control of gene expression by the pioneer factor Zelda through transient interactions in hubs. *Nature Communications*, 9(1):1–13, 2018. doi: 10.1038/s41467-018-07613-z.

M. B. Elowitz and S. Leibler. A synthetic oscillatory network of transcriptional regulators. *Nature*, 403:335 – 338, 2000. doi: 10.1038/35002125.

T. S. Gardner, C. R. Cantor, and J. J. Collins. Construction of a genetic toggle switch in escherichia coli. *Nature*, 403:339 – 342, 2000. doi: 10.1038/35002131.

A. Gorban and O. Radulescu. Chapter 3 Dynamic and Static Limitation in Multiscale Reaction Networks, Revisited. In *Advances in Chemical Engineering*, volume 34, pages 103–173. 2008. doi: 10.1016/S0065-2377(08)00003-3.

S. P. Gordon, V. S. Chickarmane, C. Ohno, and E. M. Meyerowitz. Multiple feedback loops through cytokinin signaling control stem cell number within the Arabidopsis shoot meristem. *Proceedings of the National Academy of Sciences of the United States of America*, 106(38):16529–16534, 2009. doi: 10.1073/pnas.0908122106.

J. Gruel, B. Landrein, P. Tarr, C. Schuster, Y. Refahi, A. Sampathkumar, O. Hamant, E. M. Meyerowitz, and H. Jönsson. An epidermis-driven mechanism positions and scales stem cell niches in plants. *Science Advances*, 2(1): e1500989, 2016. doi: 10.1126/sciadv.1500989.

J. Hausser, A. Mayo, L. Keren, and U. Alon. Central dogma rates and the trade-off between precision and economy in gene expression. *Nature Communications*, 10(1):68, 2019. doi: 10.1038/s41467-018-07391-8.

F. G. Helfferich. Systematic approach to elucidation of multistep reaction networks. *The Journal of Physical Chemistry*, 93(18):6676–6681, 1989. doi: 10.1021/j100355a022.

K. Ogata. *Modern Control Engineering*. Prentice Hall, 5th edition edition, 2010.

O. Radulescu, A. N. Gorban, A. Zinovyev, and V. Noel. Reduction of dynamical biochemical reactions networks in computational biology. *Frontiers in Genetics*, 3:131, 2012. doi: 10.3389/fgene.2012.00131.

J. Rombouts, A. Vandervelde, and L. Gelens. Delay models for the early embryonic cell cycle oscillator. *PLoS ONE*, 13(3):e0194769, 2018. doi: 10.1371/journal.pone.0194769.

I. T. Tokuda, O. E. Akman, and J. C. Locke. Reducing the complexity of mathematical models for the plant circadian clock by distributed delays. *Journal of Theoretical Biology*, 463:155–166, 2019. doi: 10.1016/j.jtbi.2018.12.014.
